# Digital technologies in routine palliative care delivery: an exploratory qualitative study with health care professionals in Germany

**DOI:** 10.1186/s12913-022-08802-9

**Published:** 2022-12-13

**Authors:** Susann May, Dunja Bruch, Anne Gehlhaar, Felizitas Linderkamp, Kerstin Stahlhut, Martin Heinze, Matthew Allsop, Felix Muehlensiepen

**Affiliations:** 1 Center for Health Services Research, Brandenburg Medical School, Seebad 82/83, Rüdersdorf bei Berlin, Germany; 2 Department of Cardiovascular Surgery, Heart Center Brandenburg, Brandenburg Medical School, Bernau bei Berlin, Germany; 3 Faculty of Health Sciences Brandenburg, Brandenburg Medical School, Neuruppin, Germany; 4 Department of Oncology and Palliative Medicine, Brandenburg Medical School, Immanuel Klinik Rüdersdorf, Rüdersdorf, Brandenburg Germany; 5 Department of Psychiatry and Psychotherapy, Brandenburg Medical School, Immanuel Klinik Rüdersdorf, Rüdersdorf, Germany; 6 Academic Unit of Palliative Care, Leeds Institute of Health Sciences, University of Leeds, Leeds, West Yorkshire UK; 7 AGEIS, Université Grenoble Alpes, Grenoble, France

**Keywords:** Palliative care, Digital health, Qualitative research, Telemedicine, User experience

## Abstract

**Objective:**

To explore health care professionals’ (HCPs) perspectives, experiences and preferences towards digital technology use in routine palliative care delivery.

**Methods:**

HCPs (*n* = 19) purposively selected from a sample of settings that reflect routine palliative care delivery (i.e. specialized outpatient palliative care, inpatient palliative care, inpatient hospice care in both rural and urban areas of the German states of Brandenburg and Berlin) participated in an explorative, qualitative study using semi-structured interviews. Interview data were analyzed using structured qualitative content analysis.

**Results:**

Digital technologies are widely used in routine palliative care and are well accepted by HCPs. Central functions of digital technologies as experienced in palliative care are coordination of work processes, patient-centered care, and communication. Especially in outpatient care, they facilitate overcoming spatial and temporal distances. HCPs attribute various benefits to digital technologies that contribute to better coordinated, faster, more responsive, and overall more effective palliative care. Simultaneously, participants preferred technology as an enhancement not replacement of care delivery. HCPs fear that digital technologies, if overused, will contribute to dehumanization and thus significantly reduce the quality of palliative care.

**Conclusion:**

Digital technology is already an essential part of routine palliative care delivery. While generally perceived as useful by HCPs, digital technologies are considered as having limitations and carrying risks. Hence, their use and consequences must be carefully considered, as they should discreetly complement but not replace human interaction in palliative care delivery.

**Supplementary Information:**

The online version contains supplementary material available at 10.1186/s12913-022-08802-9.

## Significance & innovation

- First exploratory study on care providers’ perspectives and experiences on digital technologies use in routine palliative care in Germany.
- Perceived benefits include acceleration of care delivery, savings in time and resources, and reduction of information losses.
- Drawbacks experienced with digital technologies include lack of personal contact, negative effects on interpersonal relationships, and socio-demographic and economic inequity.
- HCPs consider digital technology as an essential auxiliary component to support routine palliative care delivery.

## Introduction

**Fig. 1:**
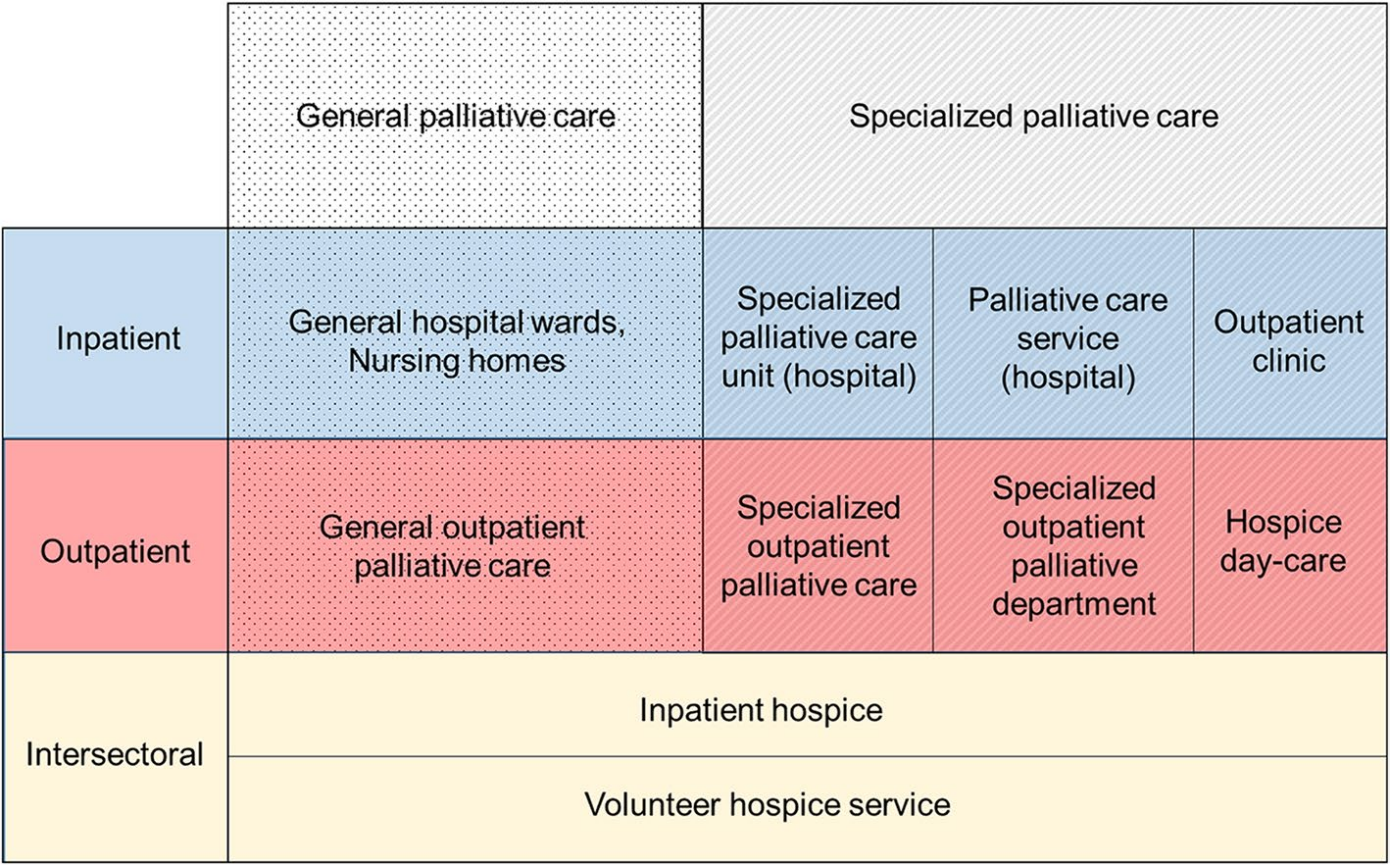
Structures of palliative care delivery in Germany (according to Simon et al. [4])

Palliative care is an approach that improves the quality of life of patients (adults and children) and their families who are facing problems associated with life-threatening illness. It prevents and relieves suffering through the early identification, impeccable assessment and treatment of pain and other problems, whether physical, psychosocial or spiritual [1]. Globally, over 57 million people are estimated to require palliative care every year including 31 million prior to and 26 million near the end of life. The majority of people who require palliative care are adults over 50 years old (67%) and at least 7% are children [2]. In Germany, it is estimated that around 765,000 people require palliative care each year [3]. Although palliative care services in Germany (Fig. 1) are considered to be at an advanced stage of integration into the health care system [2], the need for palliative care is not being met [3]. According to estimates based on analyses of medical billing data, around 400,000 patients receive palliative care in Germany each year [5], Notably underserved groups tend to include middle-aged patients [5] and those living in rural areas [3].

In recent decades, digital technologies have become increasingly prevalent in healthcare delivery [6, 7]. Digital technologies are electronic systems, devices, tools and resources that generate, store or process data [6]. A systematic meta-review, including 328 studies within 21 reviews, differentiated videoconferencing or videophone, electronic health records and telephone or mobile phone as well as online interventions, including educational websites and online courses, as the most common types of digital health interventions being used in palliative care [8]. There is evidence of predominantly positive effects of digital health interventions on training, information sharing, decision making, communication, and costs in palliative care delivery [8]. Particularly in remote regions, digital health technologies are considered an opportunity to support information exchange and access to palliative care [9, 10]. However, despite the increasing literature, evidence on the efficacy of digital technologies is lacking [9].

To realize the full potential of digital technologies in palliative care, it is critical that its development and implementation is driven by end users. Consequently, patients, relatives, healthcare professionals (HCPs), and policy makers need to be involved in developing strategies and approaches to explore digital technologies in palliative care [11–13]. In the context of Germany, increasing digitization of the health system can be evidenced through the implementation of the Digital Healthcare Act in 2019 [14]. The Act seeks to drive the use of digital technology across the health system including, for example, making all prescriptions electronic, enabling easy access to online consultations, and creating the foundation for enabling health information to be exchanged faster and more easily across care settings. However, at present, there are no studies on HCPs’ experiences and preferences of digital technology use in routine palliative care delivery in Germany, providing limited guidance to steer the future adaptation and evolution of their use in care. The aim of this study was therefore to explore the HCPs’ perspectives and preferences towards digital technology and experiences of its use in routine palliative care delivery.

## Subjects and methods

### Study design

To investigate HCPs’ perspectives and preferences towards digital technology and experiences of its use in routine palliative care delivery, we carried out an exploratory qualitative study among professionals from different settings of palliative and hospice care using semi-structured interviews.

### Participants

Participants were selected using purposive expert sampling [15], to include a heterogeneous sample inclusive of all settings of palliative care (i.e. specialized outpatient palliative care, specialized inpatient palliative care, hospice care) and professional roles (i.e. nurse, coordinator, palliative care physician, general practitioner), to participate in the study. Inclusion criteria were: professional working in palliative care, psycho-oncology or hospice care, working in a healthcare setting in Germany, and willingness to participate in the study. Participants were recruited from healthcare institutions which are clinical partners of the Psycho-oncology and Palliative Care Working Group of the Center for Health System Research at the Brandenburg Medical School, representing both rural and urban areas in the states of Brandenburg and Berlin. The potential interviewees were contacted via e-mail and invited to participate in the study. The participants did not receive financial incentives.

### Data collection

Interviews were conducted in German language by A.G., F.L., S.M., D.B. and F.M. using an open-ended interview guide that was developed to elicit participants’ perspectives on digital technology use in palliative care, including benefits, drawbacks, opportunities and risks of digital technology use. Prior to the interviews, a joint training session was conducted among the research group to ensure consistency in how the topic guide was applied and reduce the risk of potential interviewer bias. The interview guide was developed by two health service researchers (S.M., F.M.), one psychologist (D.B.) and two psychotherapy students (A.G., F.L.) in an iterative review process. Prior to commencing interviews, the interview guide was tested and refined in two face-to-face pilot interviews. No revisions were necessary. The final interview guide included the following main topics: Association with digital technology, digital technology use, and opportunities and risks of digital technology in palliative care (Supplemental Material 1). Additional open follow-up questions on specific check aspects were also included to prompt further inquiry into participants’ perspectives on interview guide topics. Sociodemographic and occupational data were collected for each participant, including age, gender, profession and setting of palliative care. To reduce the risk of infection and lessen participant burden, the interviews were conducted via telephone. The interviews took place between February and September 2021.

### Data analysis

The expert interviews were audio-recorded and transcribed verbatim. Qualitative analysis of the interviews was performed iteratively by the study team (S.M., D.B., A.G., F.L. and F.M.) based on Kuckartz’s structured qualitative content analysis [16] using MAXQDA Analytics Pro 2022, Release 22.1.0, Verbi GmbH (Berlin, Germany). Relevant text passages from the interview material were coded according to a deductive-inductive procedure. Codes were grouped into categories that merged into a coding tree, which was then discussed by the members of the study team. At this stage, data collection had already been completed. Three researchers (AG, FL, SM) independently applied the coding tree to the entire material. To ensure traceability, the application of the coding tree was validated by a member check with one interview participant. For the presentation of the results, representative quotes from the discussion transcript were selected, translated into English and included in the manuscript. The manuscript has been compiled in accordance with the Consolidated Criteria for Reporting Qualitative Research (COREQ) (Supplemental Material 2) [17].

### Ethical considerations

The study was approved by data protection officer and the ethics committee of the Brandenburg Medical School Theodor Fontane, Reference ID: E-03-20201123. All study participants received a study information pack and provided their written informed consent prior to voluntary participation. The recorded interviews were pseudonymised after transcription. The coding list is stored separately from the other study documents at Center for Health Services Research of Brandenburg Medical School Theodor Fontane. Access to the coding list is restricted to the study lead. Personal data were anonymized in the transcripts.

## Results

### Participant characteristics

Nineteen interviews were conducted and analysed until theoretical saturation was reached. Mean duration of the interviews was 51 (21–86) minutes. The mean age of the participants (*n* = 19) was 49 (range: 35–61) years. Most participants were female (13/19; 68%). Almost half of the participants were nurses (9/19; 47%). Most participants had completed advanced training in palliative care (18/19; 95%). Participants served in a variety of palliative care settings, in some cases in multiple settings at once, with professionals from specialized outpatient palliative care (SOPC) (*n* = 9), hospice care (*n* = 9) and specialized inpatient palliative care (*n* = 4) being the most frequent interview partners. Detailed characteristics of study participants are shown in Table 1. One physician in SOPC and one physician in specialized inpatient palliative care did not participate in an interview despite being contacted. Both indicated interest in the study, yet it was not possible to schedule an interview due to limited time resources. From the analysis, three themes were developed: (i) Digital technology as an enabler of coordination, patient-centred care and communication; (ii) Dissonance between digital technology use and humanness and perceived risks of digital technology in palliative care, and; (iii) Emerging uses of digital technologies to enhance care delivery.

**Table 1:** Detailed characteristics of study participants

Participant	Age	Gender	Profession	PC-Setting
P01	57	Female	Coordinator / Psycho-oncologist	Hospice / SOPC
P02	61	Male	Coordinator	SOPC
P03	43	Male	Managing Director Hospice	Hospice
P04	56	Female	Nurse	Hospice
P05	57	Female	Chief physician	Palliative care unit (hospital)
P06	44	Female	Nurse	Hospice
P07	42	Female	Geriatric nurse	Hospice
P08	58	Male	Physician	SOPC / Hospice
P09	46	Female	Geriatric nurse	SOPC
P10	42	Female	Nurse	SOPC
P11	35	Female	Nurse	Hospice
P12	46	Female	General practitioner	General outpatient palliative care
P13	38	Female	Senior physician	Palliative care unit (hospital)
P14	61	Female	Nurse	SOPC
P15	40	Male	Nurse	SOPC
P16	51	Female	Pediatric nurse	SOPC
P17	40	Male	Senior physician	Palliative care unit & oncology ward (Hospital) / SOPC
P18	55	Female	Psycho-oncologist	Oncology ward (hospital)
P19	57	Male	Chief physician	Palliative care unit / Oncology ward (Hospital)

#### Digital technology as an enabler of coordination, patient-centred care and communication

**Fig. 2:**
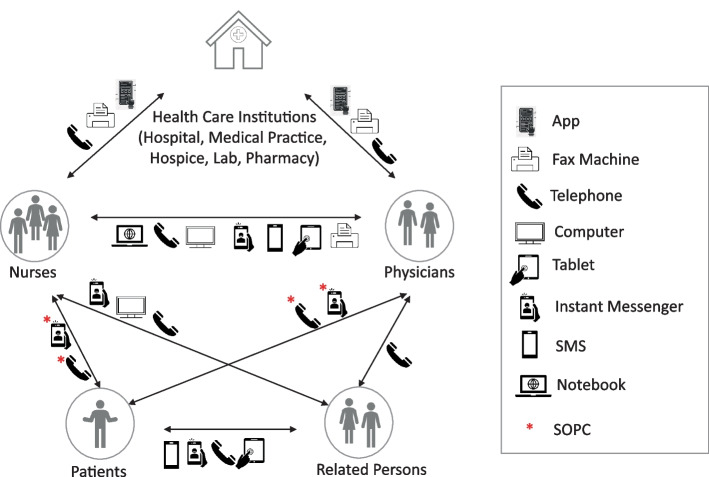
Devices used in palliative care delivery as described by the HCPs in palliative care settings

**Fig. 3:**
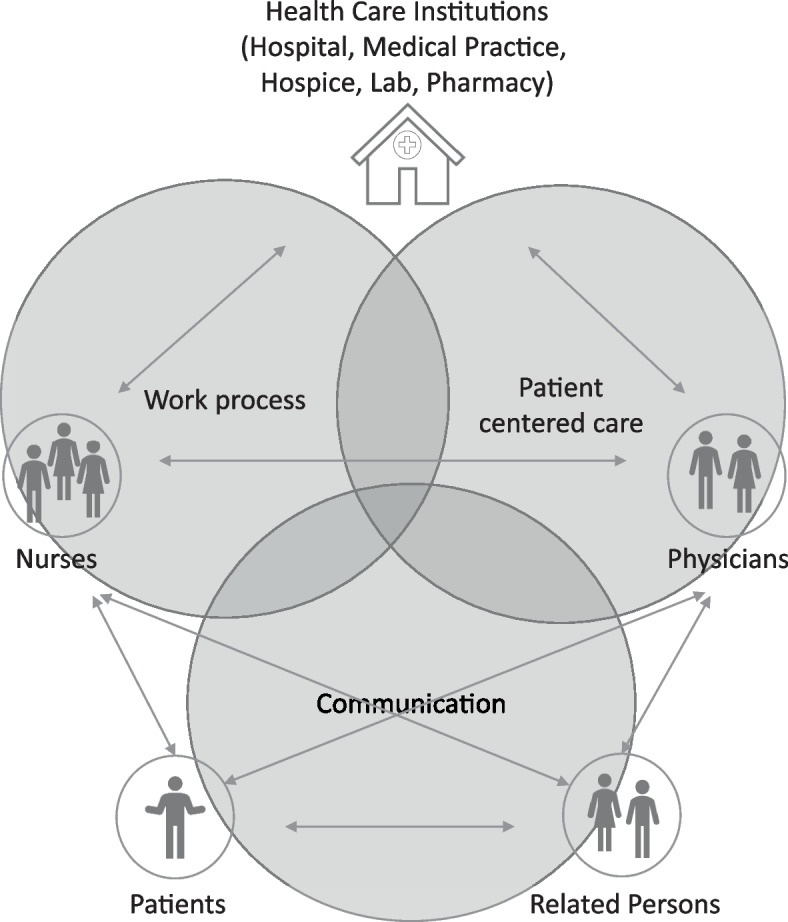
Key functions of digital technology in palliative care delivery as reported by healthcare professional participants

Participants felt that digital technologies were an integral component of routine palliative care delivery (Figs. 2 and 3).

*“This is my everyday life.* (…) *Nowadays, we can’t do without it at all. We have a computerized system. So we have an electronic patient record. My staff all have [tablets] with them. Everybody has a [smartphone] with them. I have my computer in front of me.*” *(P02, Coordinator - SOPC, S2)*

A pivotal association shared by several participants is the conversion of documentation from paper records to electronic health records. These were seen to afford participants with timely and constantly available information for palliative care professionals across inherently multidisciplinary teams:

“*Well, for me it’s just really the information - If I now enter information into the patient record, many different institutions, all the multiple professions that are involved, have access to my information and vice versa. In principle, we only have this exchange of information through digitalization. I receive orders via it and can immediately note their execution digitally. That’s what I have in mind.*” *(P06, Nurse - Hospice S4)*

Participants reported multiple digital devices used as part of palliative care delivery including smartphones, computers and notebooks, fax machines, as well as electronic health records, messenger services and short messaging and video conferencing. The main stakeholders who benefit from information exchange via digital technologies include patients and their relatives, nurses, physicians and institutions in the health care system, including hospitals, medical practices, laboratories and pharmacies. The extent of devices and technologies used varied across settings of palliative care delivery. Specifically, in outpatient care, the reported rate of digital technologies use was higher than in inpatient care. This is primarily related to decentralized delivery of care, which means that coordination of the care processes among HCPs is often only possible via phone calls or instant messaging.

“*Right now, I would say that SOPC [Specialized outpatient palliative care] would never have been possible if there hadn’t been at least telephones. So somehow the smartphone is already a pre-requisite for SOPC, I would assume* (…) *if I think of another group of terminally ill patients that I care for, those in the hospice, I observe that all these resources play a much smaller role, because the patients are much better kept by the intensive nursing care.*” *(P04, Nurse – Hospice, S8)*

The analysis of data enabled the development of three main functions of digital technology use in palliative care delivery: Coordination of the work processes, patient-centred care, and communication.

##### Coordination of the work process

Participants reported that administrative processes, such as patient documentation, are mostly digitized with the remainder expected to be in the near future. In the outpatient sector in particular, healthcare providers rely heavily on the information exchanged in the electronic patient record, as this enables them to trace the most recent treatment procedures in decentralized care delivery. Participants value the shared electronic documentation to inform and facilitate patient care, enabling more efficient work by reducing the time needed to complete documentation and through preventing mistakes and loss of patient data.

*“And the time saved. Being able to type something quickly on a tablet or laptop or whatever is very different from having to take a paper file, open it up, write it, then you misspell something. All these things-. Then you don’t read over it, then a call comes in, then there are mistakes in it that you don’t see anymore. So I actually see a lot more possibilities in this new file.” (P01, Coordinator - Hospice / SOPC, S16)*

Digital communication is valued to accelerate processes between different healthcare facilities, for example, app-based communication between providers and pharmacies resulting in patients receiving medication in a more timely and seamless way. Simultaneously, billing procedures and duty plans are also digitized.

##### Patient-centred care

The interview participants emphasized that palliative care via digital communication, such as video or phone consultations or written communication, cannot and will not replace direct contact. However, digital health technologies may support diagnostics when providers cannot be on site.

*“We actually do a bit of telemedicine via the messaging service [Name of the service]. It has a relatively high security level. Under certain circumstances, nurses can send us a photo of a wound when they are with a patient and then we can immediately reply, ‘Please look at it from the other angle again and take a few more measurements. And then we go, ‘Okay, that’s good. That should be taken care of in such and such a way.’” (P04, Nurse – Hospice, S16)*

Participants reported that digital technology enables them to access comprehensive patient information such as patient treatment and medical history. This in turn enables more efficient treatment of patients as, for example, unnecessary, duplicate examinations are avoided. Phone calls and instant messaging are also used to overcome space and time in symptom control and treatment, for example in situations where patients and relatives need immediate assistance, such as appropriate medication to reduce symptoms including pain or nausea.

*“Example: A colleague is with the patient and finds that pain medication has been maxed out. But the patient is still in pain. Then she simply calls our doctor, who is in charge. The doctor says: ‘Why don’t you give this and that, or increase the dosage?’ The colleague documents this on her computer and it is even legally secure.” (P02, Coordinator – Hospice, S3)*

*Communication.* Participants report that digital technology allows faster and more efficient information exchange.

*“It’s a relief. Getting information faster or collecting data faster, collecting data.” (P06, Nurse – Hospice, S1)*

Participants felt that information losses decrease significantly by using smartphones and electronic patient records, which in turn facilitates communication as well as information and knowledge exchange in the health care team.

*“Yes, of course, it’s totally necessary, because the organization naturally includes feedback, the short reactions via smartphone, which I mentioned earlier, or also the short message services. In principle, everybody has the opportunity to contribute to in order to prevent anyone from having to bridge a knowledge gap in a dubious situation because they simply can’t reach anyone they can ask.” (P04, Nurse – Hospice, S15)*

Nursing documentation processes in hospice care were also experienced as being shorter and faster through digitalization, which again counteracts communication losses. Information also becomes more transparent among practitioners. Shared electronic patient records in SOPC were also reported as enabling more precise instructions for medication management, which promotes patient safety.

##### Dissonance between digital technology use and humanness and perceived risks

While digital technology is increasingly being used, participants were cognizant of the potential impact of its use on relationships with patients and their relatives. Some participants deliberately avoided video- or telephone consultations with patients and relatives, as this could limit indirect communication and disrupt close relationships with their clients. Furthermore, participants expressed their reluctance to maintain the medical record system in front of patients and relatives, as they felt their time and attention should be exclusively devoted to the client. *Humanness* was mentioned in this context, which might be lost through the use of digital technology and devices.

*“It is important to understand the limits of the digital, because we humans are not digital. We humans are very analogue, very physically bound and everything we perceive, we perceive physically. And above all, we perceive things much more holistically than is often possible in digital media.” (P04, Nurse – Hospice, S5)*

According to the participants, digital communication cannot capture the various facets of the human being, which represents both a limit and a risk in the use of technology in palliative care.

*“Yes, I think it’s a very, very important point, because it’s not just about a factual level that is being conveyed. We really have many levels and a lot to do with grief and hope. And I don’t see a tear when it’s shed on the phone. That’s only when the patient sits in front of me. And I don’t see the child on the phone either, so this palliative area is particularly important there, that all these other levels - that’s also the claim of palliative medicine - this multidimensionality, that we can only do justice to it if we sitting face to face from person to person.” (P15, Nurse – SOPC, S4)*

Participants report that technology will not be able to replace the requisite human connection required in palliative care work, as technical systems cannot interact with humans as human beings can.

*“Exactly, this human to human, this… this interaction, as I said. You face each other or you see each other, you can act, a question comes up, you can give an answer. Well, a robot can too, but it doesn’t have a soul. I don’t know, so I’m rather sceptical about saying that a robot should take over everything completely and that humans don’t actually do anything with the patient anymore.” (P13, Physician – PCU, S8)*

Participants saw digital technologies as realizing their potential most when they discreetly support palliative care as an adjunct to face-to-face contact, while not taking over the centre stage or being used as an end in themselves.

*“I think at best it has great impact where patients feel well taken care of and don’t even realize digital technology is involved. Actually, my ideal is that technology shouldn’t in any way conspicuously take centre stage, but simply be lost in its willingness to serve. That the patient only notices that he is understood, his providers understand each other, the messages that come through do not contradict one another other, and everybody shares a vision and goal, and so on.” (P04, Nurse – Hospice, S20)*

Alongside the impact on delivery of care, participants also reflected on the risks and adverse effects of digital transformation, including both processes relating to the systems and their use by health professionals **(**Table 2**)**. For instance, the use of digital technologies is perceived to pose a risk to the security of patient data.

**Table 2:** Excerpt of the coding tree – Theme “Dissonance between digital technology use and humanness and perceived risks”

Category	Sub-category	Summary
Impairment of work processes	Data security	The use of digital technologies can lead to security risks and inefficiencies may arise due to technical deficits or insufficient user skills.
	Misreporting	
	Inefficiencies due to interoperability deficiencies and connectivity issues	
	Increased time consumption	
	Reduced competencies of providers and patients	
Communication restrictions	Loss of interpersonal skills	Digital technologies can affect holistic communication and human aspects can be lost.
	Digital technologies cannot capture multidimensionality of human beings	
	“Robots have no soul”	
	Digital technologies as a supplement instead of an end in itself

*“I see risks too. Of course, there is an enormous amount of data on the way, which in turn attracts others to get it or to destroy it for whatever reason. This susceptibility is of course given.” (P02, Coordinator – SOPC, S15)*

Participants also report that documents can be falsified easily or subsequently changed when being digitzed.

*“Well, in principle, if I don’t like someone, I could access my predecessor’s report with my abbreviation and change the report. […] What I’m actually getting at is that I can falsify documentation.” (P06, Nurse – Hospice, S15)*

Some devices, such as computers, notebooks, and monitors, have technical flaws, such as lack of interoperability and connectivity, or the patient record software may not work on outdated devices such as smartphones or tablets.

*“For example, using devices that are then sold again with a new guarantee, but do not exactly fit together. That was horrible and annoying to somehow make it work again. Then we had screens, for example, and the coordinator sits comfortably in her chair and wants to start working, but then the monitors don’t correspond with the computer.” (P04, Nurse – Hospice, S13)*

Specifically, in outpatient palliative care, limited network coverage to enable connection of electronic patient records but also phone calls and instant messaging impede communication. HCPs try to reach patients, relatives, or colleagues, which is not possible in rural areas due to poor mobile network coverage.

*“Connection problems of course, if you are on the road with a mobile phone, there are still enough coverage holes. If you drive around Brandenburg, you will drive from hole to hole. (…) You don’t need to try to make a phone call. But then you can’t be called either, which is the downside.” (P02, Coordinator – SOPC, S12)*

The maintenance of electronic patient records was perceived as time-consuming and cumbersome, leaving less time for patients.

*“But it’s also time-consuming, it’s a double-edged sword. Of course, I also spend a lot of time in front of the computer, which I would actually rather devote to the guests and relatives. You just have to find a healthy balance, because both are important.” (P16, Paediatric Nurse – SOPC, S2)*.

Due to limited skills and knowledge of professionals, patients and relatives, technology use might be restricted. Participants specifically pointed to the high age and severe illnesses of patients that might limit technology use.

*“On the one hand, the person I am communicating with must also be able to communicate in this way. If I have a patient who has never held a computer in their hands and I say to them, we’ll do it like this for the next few days, then I don’t have to be surprised if nothing happens.” (P02, Coordinator – SOPC, S19)*

##### Emerging uses of digital technologies to enhance care delivery

HCPs reported a wide and heterogeneous set of ideas on how digital technologies could further support palliative care delivery. For instance, digital technologies were thought to enable a wider collaboration between care providers in different settings of care, such as clinics and primary care physicians. Currently, patient records are mostly confined to sharing within existing care delivery sectors; e.g., SOPC providers can only access data in specialized outpatient care, but not inpatient care. Participants described shared access to patient records as a promising approach to overcoming siloed working by care sector and enabling HCPs from multiple sectors to store and view information with each other ensuring coordinated care and potentially reducing repeated examinations.

*“I can imagine that digital forms of communication can be better used across sectors, so that is a very interesting hurdle that we are always trying to overcome, between the clinic and the primary care doctor. In our case, also between clinic and palliative outpatient treatment team, perhaps then also hospice” (P17, Physician – PCU, S11)*

Participants indicated they had low knowledge but high acceptance of remote monitoring approaches in palliative care, such as blood pressure cuffs, pulse oximeters, glucometers, ECG/stethoscopes, spirometers, or activity trackers. Capturing this patient data might allow HCPs to continuously assess patients’ health status and evaluate the priority of a home visit.

*“I’ve made a note of the monitoring now. I will discuss this with our IT people, because I have a patient, or a few patients in mind, for whom this could be helpful.” (P01, Coordinator – Hospice/SOPC, S16)*

In the future, apps may be used in palliative care delivery to overcome existing language barriers between patients, relatives, and providers. For instance, translator apps could enable communication to identify and locate pain or help relatives to describe assistive care needs at home.

*“Of course, we sometimes have patients with a background in other languages. Not all of us speak their language. In the meantime, some have discovered that you can also use a language app. Then you can communicate simple things quite well.” (P02, Coordinator – SOPC, S14)*

Participants identified further opportunities (Table 3) for digital technologies, specifically virtual reality glasses and video conferencing systems, in the inclusion of palliative patients in family and cultural life to enhance quality of life. Currently, patients in palliative care are occasionally offered cultural activities, such as virtual museum tours, which by means of virtual reality glasses might be expanded to concerts or theatre plays, to increase their social participation and intellectual input of terminally ill patients in palliative care.

**Table 3:** Excerpt of the coding tree – Theme “Emerging uses of digital technologies to enhance care delivery”

Category	Sub-category	Summary
Support of work processes	Optimization of processes	Digital technologies support work processes, while reducing temporal (and possibly economic) burden among HCP
	Facilitation of care through joint documentation	
	Time savings through use of digital technologies	
Communication	Faster and more efficient exchange of information	Digital technologies support and intensify communication between HCP as well as between HCP and patients and their relatives, while reducing information losses.
	Fewer communication losses	
	More effective collaboration through digital meetings	
Patient-centered care	Improved quality of care	Digital technologies contribute to improved quality of care and strengthen patient-centered care by connecting health care professions/institutions and providing diagnostic and therapeutic options.

*“Yes, a tumor patient doesn’t get out of the house for the last few years and something like a trip to the museum is somehow unthinkable and so you can make it possible again! Social participation and intellectual input!” (P17, Physician – PCU, S11)*

Video conferencing could also be used to connect people in similar situations and facilitate communication and socializing.

*“Exactly. That’s where I told you about this vision (video telephony) at the beginning. That we want to deal with how we can connect people in the same life situation who may not be mobile enough to meet themselves.” (P04, Nurse – Hospice, S18)*

## Discussion

This qualitative study was conducted to explore HCPs’ perspectives, experiences, and preferences towards digital technology use in routine palliative care delivery in Germany. Digital technologies are already an essential component of routine palliative care delivery, used by HCPs across different settings, alongside by and with patients and their relatives. Digital technologies are used more widely in decentralized outpatient care delivery than in the inpatient setting, supporting providers to overcome distance and time. However, across all settings, three main functions of digital technology use in palliative care delivery include supporting coordination of work processes and care delivery, patient-centred care and communication. Although embedded in routine care, digital technologies were seen as having the potential to bridge structural gaps in palliative care delivery, allowing information to flow between healthcare institutions and providers that have not yet shared patient information. HCPs reported risks of digitalization in palliative care delivery including data security issues and administrative burden. Furthermore, digital technologies were perceived as incapable of replacing direct contact in care delivery, especially in the management of psychosocial and spiritual needs.

Our qualitative data align with the results of a meta-analysis by Finucane et al. [8]. According to our participants, digital health technology is useful, may promote patient safety, and is accepted by HCPs in palliative care delivery [8]. The HCPs confirmed electronic health records, phone communication and videoconferencing or telehealth respectively to be most relevant to their daily palliative care routines [8]. Concurrently, we found that not all digital practices reported on in recent studies are being adopted in palliative care delivery by the participants of our study. One of the four most common eHealth practices and technologies trialed in studies is mHealth [18]. Our data underlined the relevance of mobile communication in palliative care delivery, especially in the outpatient setting, although comprehensive mHealth approaches (e.g. the use of dedicated apps for accessing information or smartphone-based monitoring approaches) are not currently in use [8, 9, 18, 19]. Furthermore, existing digital practices, such as monitoring systems [20, 21] (i.e. the use of technologies to capture measurements or recordings of, for example, symptoms, daily activities, and cognitive processes) were not reported in use across any palliative care settings. Multiple factors may underpin the continuous embedding and adaptation of digital technologies over time, including at the level of the technology, the user, and more widely organizations and health system [22]. At the health professional level, participants often reported that digital technologies were being implemented intuitively (for example, the use of messenger services for diagnostics or treatment). First-hand, positive experiences appeared to determine digital health implementation in palliative care delivery. Considering the progressing digitalization in health care, practical information, and “hands-on” training modules [23, 24] that target increases in knowledge and digital skills may increase preparedness for new and emerging technologies with potential applications for palliative care (e.g. virtual reality [25] and systems for contactless continuous vital sign monitoring [26]).

Our exploratory study has raised further research desiderata. Throughout the interviews, the concept of *humanness*, specifically, the non-digital needs of human beings, the nature of human communication, and human labour considering palliative care, emerged as a topic of discussion. Serving *humanness* was also very important to palliative care providers during the Covid-19 outbreak [27]. *Humanness* might be filled with resources such as empathy, respect, tolerance, common sense, and the ability to listen and be patient as key characteristics in palliative care [28]. Further research is needed on the concept and meaning of *humanness* in palliative care delivery, and how it can surround and be preserved alongside the application of digital technologies. Furthermore, in addition to these qualitative findings, quantitative studies examining the frequency and extent of use of specific digital technologies across palliative care settings, as well as the knowledge and acceptance of their users, will be essential in informing health care providers and policy makers in planning future palliative care delivery.

To our knowledge, this is the first exploratory study on care providers’ perspectives and experiences on digital technologies use in routine palliative care in Germany. The qualitative study design allowed for an in-depth description of the participants’ perceptions of the actual use of digital technology in day-to-day palliative care delivery. Due to the open and exploratory approach providers from various disciplines and palliative care settings were able to state their personal and routine experiences with digital technology use. Our data has raised several research desiderata and it can be used to contributor key stakeholder perspectives to digital technology development and implementation with insights elicited from HCPs in routine palliative care delivery. However, limitations to the study exist. The sample included only participants from one federal state in Germany. Possibly, the recruitment of participants via the working group might have led to the involvement of sites more engaged in palliative care research. Furthermore, selection bias may have occurred through expressions of interest potentially arising from providers with an interest in digitalization. We also acknowledge that we report perceived impact of digital technologies on patient care from the health professional perspective, but those of patients and relatives have yet to be examined and reported in the research literature.

## Conclusion

Digital technology is a part of routine palliative care delivery and perceived as essential by health professionals. Digital technology has particular relevance and is commonly used to facilitate care delivery in the outpatient care setting. Central functions of digital technologies across all settings include supporting the coordination of the work processes, patient-centred care and communication. Future research is required to determine how to embed digital technologies in practice whilst ensuring the requisite elements needed to maintain humanness in care. Alongside this, there remains scope to explore the utility and acceptability of digital technologies for palliative care in Germany with a strong evidence base in other contexts (e.g. the use of mHealth approaches) alongside more emergent approaches including virtual reality.

## Supplementary Information

**Additional file 1.** Interview guide.

**Additional file 2.** Consolidated criteria for reporting qualitative research.

## Data Availability

All data relevant to the study are included in the article or uploaded as supplementary material. For further questions regarding the reuse of data, please contact the corresponding author (felix.muehlensiepen@mhb-fontane.de).
