# Supplementary material for: Digital technologies in routine palliative care delivery: an exploratory qualitative study with health care professionals in Germany
Source: BMC Health Serv Res. 2022 Dec 13;22:1516. doi: 10.1186/s12913-022-08802-9 (PMC9745710; doi:10.1186/s12913-022-08802-9)
Supplement: Supplementary file 1 — Additional file 1. Interview guide. [file 12913_2022_8802_MOESM1_ESM.docx]

**Supplemental Material 1.** Interview Guide

| **Guiding Questions** | **Check Aspects** |
| --- | --- |
| Could you please introduce yourself?  Could you please describe a typical working day? | Function, tasks, area of responsibility |
| Could you please describe: What do you associate with digital technology in palliative care? | Definition, attitude |
| Do you use digital technologies in your daily work?  For which purposes do you use digital technologies?  In which parts of palliative care do you use digital technologies? | Devices, experiences in day-to-day care delivery, preferences |
| How do digital technologies change the care of seriously ill and dying people?  What risks /opportunities do you perceive?  Who will benefit from the use of digital technologies?  Are there people or groups of people who are excluded? | Changes in care delivery, risks, opportunities, benefits, drawbacks, exclusion potential |
